# Iron availability affects West Nile virus infection in its mosquito vector

**DOI:** 10.1186/s12985-017-0770-0

**Published:** 2017-06-05

**Authors:** Jean-Bernard Duchemin, Prasad N Paradkar

**Affiliations:** CSIRO Health and Biosecurity, Australian Animal Health Laboratory, 5 Portarlington Road, Geelong, Victoria 3220 Australia

**Keywords:** West Nile virus, Mosquito, Iron, Ferritin, NRAMP, Deferoxamine

## Abstract

**Background:**

Mosquitoes are responsible for transmission of viruses, including dengue, West Nile and chikungunya viruses. Female mosquitoes are infected when they blood-feed on vertebrates, a required step for oogenesis. During this process, mosquitoes encounter high iron loads. Since iron is an essential nutrient for most organisms, including pathogens, one of the defense mechanisms for the host includes sequestration of iron away from the invading pathogen. Here, we determine whether iron availability affects viral replication in mosquitoes.

**Methods:**

To elucidate effect of iron availability on mosquito cells during infection, *Culex* cells were treated with either ferric ammonium citrate (FAC) or the iron chelator, deferoxamine (DFX). Real time RT-PCR was performed using ferritin (heavy chain) and NRAMP as a measure of iron homeostasis in cells. To determine iron requirement for viral replication, *Culex* cells were knocked down for NRAMP using dsRNA. Finally, the results were validated in *Culex* mosquito-infection model, by treating infected mosquitoes with DFX to reduce iron levels.

**Results:**

Our results show that infection of *Culex* cells led to induction in levels of ferritin (heavy chain) and NRAMP mRNAs in time-dependent manner. Results also showed that treatment of cells with FAC, reduced expression of NRAMP (iron transporter) and increase levels of ferritin (heavy chain). Interestingly, increasing iron levels increased viral titers; while reducing intracellular iron levels, either by NRAMP knock-down or using DFX, reduced viral titers. The results from *Culex* mosquito infection showed that mosquitoes treated with DFX had reduced viral titers compared with untreated controls in midgut as well as carcass 8 days pi. Saliva from mosquitoes treated with DFX also showed reduced viral titers compared with untreated controls, indicating low viral transmission capacity.

**Conclusions:**

Our results indicate that iron is required for viral replication in mosquito cells. Mosquitoes respond to viral infection, by inducing expression of heavy chain ferritin, which sequesters available iron, reducing its availability to virus infected cells. The data indicates that heavy chain ferritin may be part of an immune mechanism of mosquitoes in response to viral infections.

## Background

Mosquitoes are responsible for transmission of viruses like dengue, West Nile (WNV) and Zika viruses, which pose a huge burden on public health systems worldwide [1]. The geographic distribution of these mosquito-borne viruses is increasing due to increased travel, trade as well as global climate change [2]. Currently more than half of world’s population is at risk of getting infected with arboviruses [3].

The transmission cycle for these viruses requires alternate infections in both competent vector and susceptible vertebrate hosts [4]. Female mosquitoes transmit viruses to these vertebrate hosts during blood feeding. Other than nutrients, like proteins, which are required by females for oogenesis [5], vertebrate blood also contains high levels of iron, which is used by mosquitoes for egg development to produce viable offspring [6]. This high level of iron in the blood meal is provided as hemoglobin in erythrocytes, and as ferric-transferrin. Although most of the heme iron is excreted by the mosquito, iron from ferric-transferrin is highly absorbed [7].

Iron is an essential element for most of living organisms, important in many cellular functions. Iron-containing proteins are important for energy metabolism as well as immunity and signaling pathways [8]. On the other hand, free iron is highly reactive and toxic, due to formation of free radicals. Hence mammals have developed a highly regulated process of iron homeostasis [9]. In mammals, cellular available iron is regulated by iron transporters [10]. Membrane iron transporter, NRAMP2 (Natural Resistance-Associated Macrophage Protein), mediates iron import while ferroportin is the only known iron exporter. Increase in intracellular iron leads to decrease in iron importer (NRAMP2), and increase in ferroportin (iron exporter). Free iron in mammals is sequestered, using iron-binding proteins, like ferritin within the cell and transferrin in the blood. Production of these proteins is also under the control of free iron [11]. Similarly, mosquitoes are also protected from oxidative stress by storing iron within ferritin [12].

Most of the proteins involved in iron metabolism are conserved between mammals and insects. However there are certain differences. In mosquitoes, ferritin is expressed throughout the life cycle and increases following a blood meal [13]. Similar to vertebrates, mosquito ferritin also consists of 24 subunits of heavy and light chain polypeptides, both of which respond to blood-feeding as well as by iron treatment [6]. In mammals, ferritin binds to intracellular free iron and sequesters it [9]. Interestingly, in mosquitoes, ferritin is secreted in response to iron treatment [12], while in mammals is rarely secreted in the blood. A recent report has indicated that in mosquito midgut epithelium, iron is loaded onto ferritin and, unlike mammals, is secreted into hemolymph regulating intracellular iron overload [14].

In contrast with mammals, mosquito genome contains only a single gene of NRAMP [15], which imports iron into cell and has also been recently implicated in alphaviral entry [16]. NRAMP is also under the control of intracellular iron levels, with low intracellular iron leading to higher expression.

Since iron is an essential nutrient for most organisms, including pathogens, one of the defense mechanisms for the host includes sequestration of iron away from the invading pathogen [17]. Iron acquisition proteins are virulence factors for many bacterial species [18]. Previous studies have shown that bacterial infections lead to increases in hepcidin [19], iron regulator protein, in mammals, which by degradation of ferroportin prevents export of iron out of the cell [20]. This leads to sequestration of iron in intracellular compartment. During infection, in mammals, this induced hypoferremia may limit growth of extracellular bacteria, however cellular iron sequestration increases growth of intracellular bacteria like *Chlamydia psittaci, C. trachomatis*, and *Legionella pneumophila* [21]*.* Although viruses do not require iron themselves, infected cells need iron for replication and in turn assembly of virus particles. Previous studies have indicated decrease in intracellular iron load affects Human Immunodeficiency Virus [22] and Hepatitis C Virus [23] replication. *In vitro* studies have shown that iron reduces HCV replication through its effect on a number of host genes, including eukaryotic translation initiation factor 3, which is involved in translation [24]. Iron also serves as a co-factor for number of genes essential for replication.

As in mammals, transferrin expression in mosquitoes has been shown to increase during infection with bacteria and during encapsulation of filarial worm, an extra-cellular pathogen [25]. Transferrin is considered as a part of the immune defense system as an acute-phase protein. Much less is known about ferritin and mosquito infection. Previous RNA-Seq data from our lab has shown that ferritin, light chain, was down regulated in Culex cells after WNV infection [26].

Since mosquitoes are subjected to substantial amount of iron while they take up viruses during blood feeding, it is not known whether manipulation of iron can lead to changes in viral load and in their capacity as vector. Virus also induces immune reaction in mosquitoes and whether iron metabolism proteins play a role in immunity is not explored. Here we show that mosquitoes infected with WNV, display increased expression of ferritin and NRAMP. We also show that reducing availability of intracellular iron, either by knockdown of NRAMP or by treatment with deferoxamine, an iron chelator, led to significant decrease in viral load in mosquitoes, by decreasing viral replication. Our results show that iron metabolism in mosquitoes plays an important role in virus transmission and provides a possible target for reducing mosquito-borne viral disease burden.

## Methdos

### Cell culture and virus propagation

Hsu (*Culex quinquefasciatus*) cells were maintained at 28 °C in Leibovitz’s L-15 medium (Gibco #11415) containing 10% tryptose phosphate broth solution, 15% heat-inactivated fetal bovine serum, and 1% penicillin-streptomycin solution. West Nile virus (NY99–4132 strain) was used for the study. C6/36 (*Aedes albopictus*) cells were maintained in RPMI medium at 28 °C and were used to propagate the virus. Vero cells maintained in EMEM at 37 °C were used for plaque assays.

### Real time RT-qPCR

Total RNA was extracted from cells using RNeasy kit (Qiagen) according to the manufacturer’s protocol. Reverse transcription was performed with random hexamer primers using the First Strand Synthesis kit (Invitrogen). Real-time RT-qPCR was performed using following gene-specific primers. Ferritin, heavy chain: Forward = TCAAGCTGATCGAGTACGCC, Reverse = CATCCTCCAGAGCGGACAAT. NRAMP: Forward = CCCTGTAAGCATCGTGGGTT, Reverse = TCTTGCACGGTGCTAACGAT.

As an internal control, real-time RT-qPCR was also performed using the housekeeping gene, RpL32 (Forward: CGAGCAGCAGTTGCCCAGCT, Reverse = GCTGAAGGGGTCCGGGTTGC). For WNV NS1: Forward = ATCGCGCTTGGAATAGCTTA, Reverse: GACAGCCGTTCCAATGATCT. The control was set arbitrarily at 1 and fold-increase over control was calculated by the ΔΔCt method. The experiments were conducted at least three times, each in triplicates. The results were plotted in graph format as mean ± SD.

### Plaque assays

Plaque assays were performed as previously described [27]. In brief, supernatant media from cells infected with WNV (10-fold dilutions) were added onto confluent Vero cell monolayers in 6-well plates. After 1 h incubation at 37 °C, the cells were overlaid with medium containing agar. Plaques formed within 4 days pi were counted and the results were plotted graphically. The experiments were conducted at least twice, each with duplicates.

### Iron and deferoxamine treatment

Cells were treated with 100 μM of ferric ammonium citrate (FAC, Sigma-Aldrich) with or without WNV infection. To determine effect on entry of virus, cells were treated with FAC (100 μM) 2 h before infection with WNV. Cells were treated with 20 μM of deferoxamine mesylate (DFX, Sigma-Aldrich) with or without WNV. For cell viability assay, cells were treated with various increasing concentrations of DFX (0, 10, 100 and 1000 μM). For mosquito infections, DFX was added to the blood (10 μM final) used for feeding mosquitoes with or without virus infection.

### Cell viability assay

Cell viability assay was performed using trypan blue exclusion staining followed by counting cells using hemocytometer. Cell viability was calculated as the number of viable cells divided by total number of cells, with control set at 100%.

### dsRNA knockdown

Gene-specific dsRNA (~400 nt) were prepared using the MEGAscript RNAi kit (Thermo Fisher Scientific) according to the manufacturer’s protocol. Following primers were used to prepare dsRNA against *Aedes* NRAMP (XP_001656702). Forward: GAATTAATACGACTC ACTATAGGGAGAACATTACCGACAAACCGACC and Reverse: GAATTAATACGACTCACTATAGGGAGAGCCAGCTTGCGCTTAATCGT. dsRNAs were transfected into Hsu cells using Cellfectin according to a previously described protocol [27]. dsRNA against green fluorescent protein (GFP) was used as a knock-down specificity control.

### Mosquito experiments

*Culex annulirostris* mosquitoes were maintained in a diurnal cycle (12 h/12 h) with temperatures alternative between 23 and 26 °C and 65% humidity. Three to 5 day-old female mosquitoes (n = 20) were blood-fed on chicken skin membranes with WNV (1.13×10∧6 pfu/ml) with or without deferoxamine (10 μM) and the mosquitoes were incubated at 25 °C and 65% humidity in an environmental cabinet (Thermoline Scientific, Smithfield, Australia) with a wet cotton pad (10% sucrose solution) provided daily as a food source. At 8 days post-infection, saliva was collected in capillary tubes containing 5 μl FCS for 10 min using a protocol described previously [26]. Midguts were dissected at 2 or 8 days post-infection and homogenized using a bead-beater. RNA was extracted using the RNeasy kit from the midgut and carcass of individual mosquitoes and was used for real-time RTqPCR as described above. Saliva from each mosquito was diluted in 300 μl of L-15 medium and was used to determine viral titer by plaque assay as described above.

### Statistical analysis

In vitro experiments were conducted in triplicates and means from each experiment were used to calculate Standard deviation (SD) and data analyzed using the unpaired Student’s *t*-test for single mean comparisons.

## Results

**Fig. 1:**
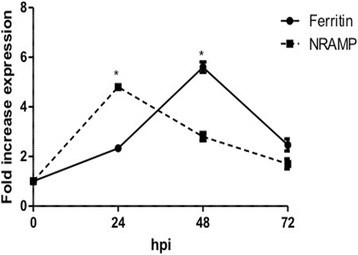
WNV infection changes ferritin and NRAMP expression in *Culex* cells. *Culex* (Hsu) cells were infected with WNV (MOI of 1). Total RNA was collected from cells at 0, 24, 48 and 72 hpi. Real-time RT-qPCR was performed using *Culex* ferritin (heavy chain) and NRAMP primers. RpL32 primers were used as an internal control. Error bars represent standard errors from means of three separate experiments with assays performed in triplicate (Student’s *t*-test *p < 0.05, comparing with 0 time point)

Previous research has shown that iron availability plays a major role during the infection process. Initially, to determine whether mosquito iron metabolism is influenced by viral infection, *Culex* cells were infected with WNV (multiplicity of infection, MOI of 1) and real time RT-PCR was performed to determine expression levels of iron storage protein, ferritin (heavy chain) and iron transporter, NRAMP. The results showed time-dependent increase in mRNA levels of ferritin after WNV infection, peaking at 48 h post-infection (6-fold increase), followed by decrease at 72 hpi (Fig. 1). Expression of NRAMP showed early increase (5-fold) at 24 hpi, followed by decrease at 48 and 72 hpi. The results indicate that viral infection leads to changes in iron metabolism.

**Fig. 2:**
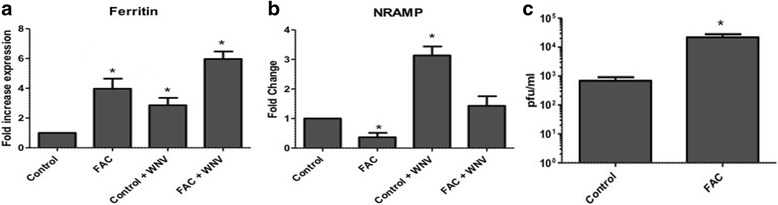
Iron treatment increases viral titers in *Culex* cells. *Culex* cells were infected with WNV (MOI of 1) with/without iron (FAC, 100 uM) treatment. As a control, uninfected cells were used. Total RNA and supernatant medium were collected at 48 hpi. Real-time RT-qPCR was performed using *Culex* ferritin (heavy chain) (**a**) and NRAMP (**b**) primers. RpL32 primers were used as an internal control. Error bars represent standard errors from means of three separate experiments with assays performed in triplicate (Student’s *t*-test *p < 0.05, comparing with control untreated cells). **c** Viral titer estimation by plaque assays conducted on the supernatant media collected from cells treated as in (**a**). (Student’s *t*-test *p < 0.05, comparing with control infected cells)

To determine whether changes in iron availability have an impact on virus replication in mosquitoes, *Culex* cells infected with WNV were treated with ferric ammonium citrate (FAC). As a control cells were either left uninfected or untreated with FAC. Cell culture supernatant and total RNA from cells were collected at 48 hpi. Real time RT-PCR results showed increased expression of ferritin (heavy chain) in cells treated with FAC (4-fold) compared with controls, with higher increase (6-fold) in infected cells treated with FAC (Fig. 2a). The result also showed 2-fold decrease in expression of NRAMP as a response to FAC treatment, with or without WNV infection (Fig. 2b). Viral titration performed by plaque assay showed increased viral titers from cells treated with FAC (2.2 × 10^4 pfu/ml) compared with untreated cells (7 × 10^2 pfu/ml) (Fig. 2c), indicating proviral effect of iron.

**Fig. 3:**
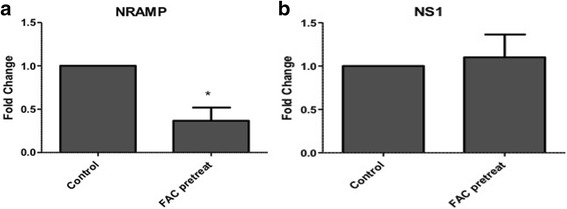
Culex NRAMP does not act as WNV receptor Culex (Hsu) cells were pretreated with FAC (100 uM) for 2h before infection with WNV. Total RNA was collected 3 hpi and real time RT-PCR was performed using Culex NRAMP (**a**) and WNV NS1 (**b**) primers. RpL32 primers were used as an internal control. Error bars represent standard errors from means of three separate experiments with assays performed in triplicate (Student’s *t*-test *p < 0.05, comparing with control infected cells)

NRAMP has been previously implicated in alphaviral entry in mosquitoes and mammals [16]. To determine whether change in iron level leads to change in viral entry as a result of expression of iron importer, NRAMP, *Culex* cells were treated with FAC 2 h before infection with WNV. Total RNA from cells was collected 3 hpi and real time RT-PCR for WNV NS1 was performed to determine viral entry into cells. Results showed that there were no significant changes in NS1 levels in cells treated with FAC compared with control, indicating that iron availability does not impact viral entry (Fig. 3b), as was shown in Rose et al. [16] for Sindbis virus. As a control, real time RT-PCR for NRAMP showed > 2-fold decrease in cells treated with FAC (Fig. 3a), indicating that there was in fact decrease in NRAMP level after 2 h pretreatment as seen before [28].

**Fig. 4:**
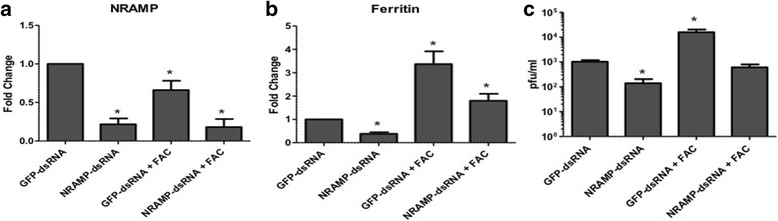
Reducing intracellular iron affects viral titers. Culex (Hsu) cells were transfected with dsRNA against Culex NRAMP 24 h before infection with WNV (MOI of 1). GFP-dsRNA was used as a negative control. Cells were also treated with FAC (100 uM). Total RNA and media supernatant were collected 48 hpi. Real time RT-PCR was performed using NRAMP (**a**) and ferritin (heavy chain) (**b**) primers. RpL32 primers were used as an internal control. Error bars represent standard errors from means of three separate experiments with assays performed in triplicate (Student’s *t*-test *p < 0.05, comparing with GFP-dsRNA cells). **c** Viral titer estimation by plaque assays conducted on the supernatant media collected from cells treated as in (**a**). (Student’s *t*-test *p < 0.05, comparing with control infected (GFP-dsRNA) cells)

To further determine significance of iron availability during viral infection in mosquitoes and to reduce intracellular iron, *Culex* cells were transfected with dsRNA against NRAMP to knock-down the gene 24 h before infection with WNV along with FAC. As a control, cells were either transfected with dsRNA against GFP or left untreated (no FAC). Total RNA from cells and cell media supernatant were collected 48 hpi. Real time RT-PCR results showed significant decrease (~80%) in expression of NRAMP (Fig. 4a), indicating efficient knockdown. Results also showed a 50% decrease in ferritin expression (Fig. 4b) compared with control (GFP dsRNA), indicating decreased intracellular iron. Plaque assay results showed that viral titers were significantly lower in cells silenced for NRAMP (1.4 × 10^2 pfu/ml) compared with control (1 × 10^3 pfu/ml) (Fig. 4c). Viral titers increased with the presence of FAC (1.6 × 10^4 pfu/ml), however in cells silenced for NRAMP, viral titers were significantly lower (6.1 × 10^2 pfu/ml).

**Fig. 5:**
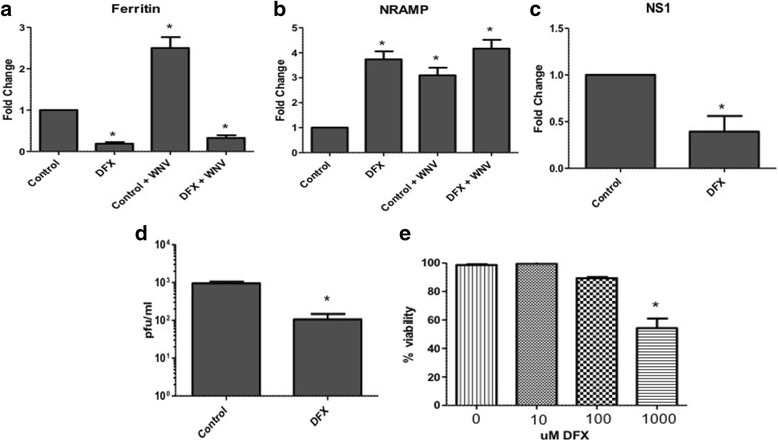
Iron chelator, deferoxamine, reduces viral titer in Culex cells Culex (Hsu) cells were infected with WNV with/without deferoxamin (DFX, 10 uM). As a control cells were either uninfected and/or treated with DMSO (diluent). Total RNA and media supernatant were collected 48 hpi. Real time RT-PCR was performed using ferritin (heavy chain) (**a**), NRAMP (**b**) and WNV NS1 (**c**) primers. RpL32 primers were used as an internal control. Error bars represent standard errors from means of three separate experiments with assays performed in triplicate (Student’s *t*-test *p < 0.05, comparing with control cells). **d** Viral titer estimation by plaque assays conducted on the supernatant media collected from cells treated as in (**a**). (Student’s *t*-test *p < 0.05, comparing with control infected cells). **e** Cell viability was measured from Culex (Hsu) cells treated with different concentrations (0, 10, 100 and 1000 uM) of DFX for 48 h using trypan blue exclusion

In order to determine whether reducing iron availability impacts on viral infection and replication in mosquitoes, *Culex* cells were treated with iron chelator, deferoxamine (DFX, 10uM), with or without WNV infection. Cell media supernatant and total RNA from cells were collected at 48 hpi. Real time RT-PCR showed reduced (~75%) expression of ferritin (heavy chain) in cells treated with DFX (with and without infection) compared with controls (Fig. 5a). The results also showed increased expression (3.5-fold) of NRAMP in these cells treated with DFX compared with control (Fig. 5b). Viral replication was measured by performing real time RT-PCR using WNV NS1 primers and the results showed significant reduction (65%) in NS1 levels in cells treated with DFX compared with control (Fig. 5c). Plaque assay performed on media supernatant showed reduced viral titers from cells treated with DFX (1 × 10^2 pfu/ml) compared with control (9.5 × 10^2 pfu/ml) (Fig. 5d), indicating that reducing available intracellular iron reduces viral replication in cells. Experiments were also performed to determine cell viability after DFX treatment. The results showed cell viability was reduced at 1 mM concentration, however there was no change in viability of cells at concentrations used for experiments (Fig. 5e), indicating that reduction in viral titer was not due to cell death.

**Fig. 6:**
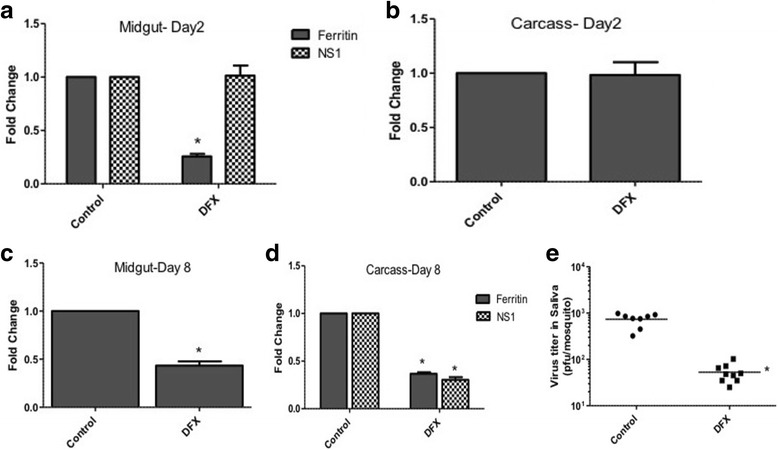
DFX treatment reduces viral titers in Culex mosquitoes Female Culex annulirostris mosquitoes were blood-fed with WNV for 1 h, with or without deferoxamine (10 uM). Mosquito were dissected at 2 and 8 days pi to collect midgut and carcass and were processed for RNA extraction. Real time RT-PCR was performed using ferritin (heavy chain) and WNV NS1 primers on day 2 midgut (**a**). Real time RT-PCR was also performed using ferritin primers on day 2 carcass (**b**), day 8 midgut (**c**) and day 8 carcass (**d**). Real time RT-PCR was also performed using WNV NS1 primers on day 8 carcass (**d**). RpL32 primers were used as an internal control. Error bars represent standard errors from means of two separate experiments with assays performed in triplicate (Student’s *t*-test *p < 0.05, comparing with infected control mosquitoes). **e** Viral titer estimation conducted by plaque assay in mosquito saliva mixed with cell culture medium. Viral titers from individual saliva samples were plotted on the graph (Student’s *t*-test *p < 0.05, comparing between with infected control mosquitoes). Control N=8; DFX N=10

To validate these findings in a mosquito infection model, female *Culex annulirostris* mosquitoes, which were previously shown to transmit WNV [29], were fed with blood containing WNV with or without DFX. Saliva was collected from mosquitoes 8 days pi to determine virus titers. Mosquito were dissected at 2 and 8 days pi to collect midgut and carcass (remainder of the mosquito after midgut was removed) and were processed for RNA extraction. Real time RT-PCR showed reduced expression of ferritin (heavy chain) in mosquito midguts at day 2 pi (70%) (Fig. 6a-b) and in mosquito midguts (60%) (Fig. 6c) and carcasses (70%) (Fig. 6d) at day 8 pi. The results also showed that WNV NS1 levels in mosquito midgut were comparable at day 2 pi (Fig. 6a), demonstrating lack of effect of DFX on viral entry from the blood meal. However, at day 8 there was significant reduction in NS1 levels in carcasses from mosquitoes treated with DFX (Fig. 6d). Plaque assays were performed on saliva collected at day 8 pi and results showed significantly reduced viral titers in saliva collected from mosquitoes treated with DFX (5.3 × 10^1 pfu/mosquito) compared with untreated control (7.4 × 10^2 pfu/mosquito) (Fig. 6e).

## Discussion

Transmission of arboviruses, like dengue and West Nile virus, require alternate transmission between invertebrate mosquitoes and vertebrate hosts [4]. Female mosquitoes require blood as a source of proteins and other nutrients, including iron, during egg development [5]. Iron is an essential nutrient and due to its redox activity, plays a significant role at the active center of various enzymes in cells. On the other hand, due to the same properties, iron in excess is also toxic to cells, by production of reactive oxygen species. Hence, levels of iron in cell are highly regulated, by import of iron and its storage [8, 9]. As in mammals, mosquito genome also encodes for ferritin, transferrin and NRAMP, which play a role in iron homeostasis [13]. Previous studies have reported that ferritin is the major protein for sequestering as well as trafficking iron in insects. Mosquito ferritin consists of heavy chain (HCH) and light chain (LCH). In *Aedes* mosquitoes, the HCH subunits predominate in hemolymph, are present in sugar-fed adults and increase dramatically with blood feeding. In contrast, the LCH is not found in sugar-fed adults and is only modestly increased in response to blood feeding [30]. Here, we show that HCH ferritin mRNA was significantly induced in response to iron as well as viral infection, confirming that *Culex* HCH is under control of iron availability. The results also suggest that viral infection also leads to induction of HCH expression, indicating possible involvement in defense mechanisms. NRAMP serves as an iron importer in mosquitoes, which is also under the control of iron availability, with increased expression in low iron situations [15]. Our results confirm that *Culex* NRAMP mRNA was downregulated by iron treatment.

Mosquitoes are exposed to large amount of iron in the blood meal in the form of heme-bound and ferric-transferrin iron. Although a large percentage of heme-iron is excreted and not absorbed, mosquitoes still absorb large percentage of transferrin-bound iron [7]. Previous work has included a global analysis of heme-regulated mosquito transcriptional changes and observed an iron- and reactive oxygen species (ROS)-independent signaling induced by heme that comprised genes related to redox metabolism [31]. Heme triggered changes in the expression of energy metabolism and immune response genes, altering the susceptibility towards bacteria and dengue virus. Here we show changes in iron metabolism in mosquito cells after viral infection, and reveal how manipulation of available intracellular iron affects viral replication in mosquitoes.

Our results, here, show that infection of mosquito cells with WNV, leads to upregulation of expression of heavy chain ferritin. Previous studies have shown that ferritin, in mammals as well as mosquitoes, is induced in response to bacterial infection [17]. Pathogens, like bacteria, also require iron for their growth. In fact, iron acquisition proteins are virulence factors for many bacterial species [18]. Iron is highly insoluble and has low bioavailability despite its abundance, leading to fierce competition between a host and its pathogens for its acquisition. For host organisms, one of the defense strategies is to sequester iron during infection to compromise viability of invading pathogens. Previous work done in our laboratory has shown light chain ferritin may be down-regulated in *Culex* cells after WNV infection [26]. Here we show that in mosquitoes, blood feeding with WNV leads to increase in heavy chain ferritin mRNA. Our *in vitro* results also show that WNV infection leads to an increase in heavy chain ferritin expression. This indicates the possibility that mosquito heavy chain ferritin expression is under control of not just iron, but also regulated by factors activated by infection, possibly as a part of immune response. This has been previously proposed by Geiser et al. 2013 [32], as a result of bacterial infection in mosquitoes. It appears that ferritin (heavy chain), in insects, may work as an immune gene, activated in response to infection, to sequester available iron away from invading pathogen. Our results indicate that light chain ferritin may not play a role during infection process.

Our results show that iron supplemented mosquito cells had higher viral titers compared with control cells. Previous studies have shown that iron availability plays a major role in growth of intracellular bacteria in mammals [21]. Our results indicate that availability of intracellular iron (as determined by increased ferritin) leads to increased viral replication. Iron serves as a co-factor for a number of host proteins involved in transcription and translation. Because the process of viral genome replication and protein synthesis requires iron, it is expected that increased iron availability will lead to increased viral replication. In fact, previous studies have indicated that increased iron availability increases replication of viruses like hepatitis C virus [23] and HIV [22]. Although exact mechanism of iron requirement in mosquito viral replication is not known, our results indicate that similar processes may be at play during mosquito infection with arboviruses.

Deferoxamine (DFX) is an iron chelator, which has been used to treat acute iron poisoning as well as hemochromatosis in patients [33, 34]. Previous studies have shown that intracellular bacterial growth can be blocked by treatment of cells with DFX [21, 35]. In one retrospective study, thalassemia patients infected with HIV, who received higher doses of iron chelator survived longer, indicating reducing iron levels are beneficial [36]. A number of in vitro studies have shown that treatment of cells with iron chelators inhibits viral replication, including herpes simplex virus [37], hepatitis B virus [38] as well as hepatitis C virus [24]. Since mosquitoes are subjected to high concentration of iron during their blood meal along with virus, mosquitoes have to deal with sequestering the iron to prevent its toxic effects as well as to prevent its availability for pathogen growth. Our results show that mosquitoes induce heavy chain ferritin levels in response to blood meal and viral infection. Treatment of cells with DFX led to chelation of iron (as indicated by reduced ferritin levels and increase NRAMP level), which further led to reduced viral titers. The results were also validated in mosquito infection model. Feeding mosquitoes with DFX containing infected blood led to reduction in viral titers in the saliva of these mosquitoes by approximately 1 log. The significance of this decrease on WNV transmission and epidemiology remains to be seen; however, the results indicate a need of iron for viral replication in mosquitoes.

Our results show that mosquitoes are subjected to high iron levels during blood feeding, which is important for viral replication. The data presented here emphasize iron requirement during viral replication and may shed light on role played by iron homeostasis proteins, heavy chain ferritin and NRAMP, in defense. The study opens up new area of research in understanding the role of iron during mosquito infection. The results may have larger implications in understanding mosquito immune response as well as novel strategies to curb mosquito-borne viral transmission.

## Conclusions

Arboviruses require transmission of virus by mosquitoes during blood feeding. Vertebrate blood is rich in iron along with other nutrients, required for oogenesis. It is not known whether iron availability affects infection and viral replication in mosquitoes. This study confirms that iron metabolism plays a major role during transmission of arboviruses. Reducing iron availability affects viral replication and titers in mosquitoes. The results also indicate that heavy chain ferritin may play a role in a mosquito defense mechanism by sequestration of iron. Overall, this work highlights importance of studying iron metabolism and homeostasis during infection in mosquitoes.

## Abbreviations

DFX: Deferroxamine
FAC: Ferric ammonium citrate
HCV: Hepatitis C virus
HIV: Human immunodeficiency virus
MOI: Multiplicity of infection
NRAMP: Natural resistance-associated macrophage protein
WNV: West Nile virus
